# Emerging Monogenic Complex Hyperkinetic Disorders

**DOI:** 10.1007/s11910-017-0806-2

**Published:** 2017-10-30

**Authors:** Miryam Carecchio, Niccolò E. Mencacci

**Affiliations:** 1 Molecular Neurogenetics Unit, IRCCS Foundation Carlo Besta Neurological Institute, Via L. Temolo 4, 20126 Milan, Italy; 2 Department of Pediatric Neurology, IRCCS Foundation Carlo Besta Neurological Institute, Via Celoria 11, 20131 Milan, Italy; 3 Department of Medicine and Surgery, PhD Programme in Molecular and Translational Medicine, Milan Bicocca University, Via Cadore 48, 20900 Monza, Italy; 4 Department of Neurology, Northwestern University, Feinberg School of Medicine, Chicago, IL 60611 USA; 5 Department of Molecular Neuroscience, UCL Institute of Neurology, London, WC1N 3BG UK

**Keywords:** Hyperkinetic, Movement disorders, Genetics, Next-generation sequencing, Epilepsy

## Abstract

**Purpose of Review:**

Hyperkinetic movement disorders can manifest alone or as part of complex phenotypes. In the era of next-generation sequencing (NGS), the list of monogenic complex movement disorders is rapidly growing. This review will explore the main features of these newly identified conditions.

**Recent Findings:**

Mutations in *ADCY5* and *PDE10A* have been identified as important causes of childhood-onset dyskinesias and *KMT2B* mutations as one of the most frequent causes of complex dystonia in children. The delineation of the phenotypic spectrum associated with mutations in *ATP1A3*, *FOXG1*, *GNAO1*, *GRIN1*, *FRRS1L*, and *TBC1D24* is revealing an expanding genetic overlap between epileptic encephalopathies, developmental delay/intellectual disability, and hyperkinetic movement disorders,.

**Summary:**

Thanks to NGS, the etiology of several complex hyperkinetic movement disorders has been elucidated. Importantly, NGS is changing the way clinicians diagnose these complex conditions. Shared molecular pathways, involved in early stages of brain development and normal synaptic transmission, underlie basal ganglia dysfunction, epilepsy, and other neurodevelopmental disorders.

## Introduction

Hyperkinetic movement disorders are a heterogeneous group of neurological disorders defined by an excess of involuntary movement production.

Based on the clinical phenomenology, hyperkinetic movement disorders are classified in different clinical entities, including among others dystonia, chorea, and myoclonus. Dystonia is characterized by sustained or intermittent muscle contractions causing abnormal, often repetitive, and patterned movements and/or postures. Chorea features continuous and brief involuntary movements, typically flowing from one body part to another in an unpredictable fashion in terms of timing, speed, and direction. Myoclonus defines shock-like involuntary jerks caused by rapid muscle contractions [1].

The etiology of these disorders is often genetically determined, especially in pediatric cases, and mutations in a rapidly growing number of genes have been causally linked to hyperkinetic movement disorders.

Traditionally, a detailed characterization of the predominant movement disorder observed on examination would lay the bases for subsequent investigations, including targeted genetic analysis of mutations in genes that are known to be associated with a specific movement disorder.

However, there are several pitfalls that can make establishing a precise diagnosis on clinical grounds a true challenge. First, multiple hyperkinetic movement disorders are frequently observed together in the same patient with a considerable degree of overlap, which makes defining the predominant type of movement disorder very difficult. Second, the clinical presentation of hyperkinetic movement disorders is often complex and highly variable, which may lead different neurologists to label differently the same movement disorder. Finally, additional neurological features (including intellectual disability (ID), epilepsy, spasticity, ataxia, and structural abnormalities of the brain) are often observed, in variable combinations, especially in cases with pediatric onset.

Hence, it is not surprising how the diagnostic work-up for complex hyperkinetic movement disorders may easily turn into long and painful diagnostic odysseys for patients and their families.

Importantly, the advent of next-generation sequencing (NGS) is rapidly changing the way clinicians diagnose and identify these conditions. Diagnostic approaches based on NGS technologies (i.e., targeted gene sequencing panels or whole-exome sequencing) are progressively becoming a first-line asset in the diagnostic pipeline for these complex disorders, partially bypassing the difficulties of the clinical assessment.

To increase awareness of these individually rare conditions, in this review, we will summarize the main clinical and genetic features of the genetically determined complex hyperkinetic movement disorders identified in the last 5 years (summarized in Table 1).

**Table 1:** Synopsis of the most relevant genes associated with complex hyperkinetic movement disorders

Gene	Main associated phenotype	Gene product	Inheritance	Age of onset	Diagnostic clues
*ADCY5*	*ADCY5*-related chorea	Adenylate cyclase 5	AD/de novo	Infancy to childhood	Axial hypotonia and delayed milestones Diurnal and sleep-related MD exacerbations Dystonia and myoclonus prominent in some cases
*PDE10A*	*PDE10A*-related chorea	Phosphodiesterase 10A	De novo/AD/AR	Infancy to childhood	Delayed motor-language milestones and dysarthria in recessive cases MRI: symmetrical T2-hyperintense bilateral striatal lesions in cases with heterozygous de novo mutations
*FOXG1*	Congenital Rett disease	Forkhead Box G1	De novo	Infancy to early childhood	Severe ID, absent language, acquired microcephaly MRI: corpus callosum aplasia/hypoplasia, delayed myelination, simplified gyration
*ARX*	Early infantile epileptic encephalopathy-type 1; X-linked mental retardation	Aristaless-related homeobox protein	XL	Infancy	Ohtahara/West syndrome, severe mental retardation, generalized dystonia/dyskinesias with recurrent status dystonicus
*STXBP1*	Early infantile epileptic encephalopathy-type 4	Syntaxin-binding protein 1	De novo	Early infancy to childhood	Onset of seizures within one year of age. Developmental delay, ID, autistic-like features, ataxia with or without dyskinesias/dystonia
*SYT1*	Severe motor delay and intellectual disability	Synaptotagmin-1	De novo	Infancy	Severely delayed motor development without seizures
*UNC13A*	Congenital encephalopathy with dyskinesias	Unc-13 homolog A	De novo	Congenital	Developmental and speech delay; ID, congenital dyskinesias with intention tremor, rare febrile seizures
*GNAO1*	Early infantile epileptic encephalopathy type 17/Ohtahara syndrome	Gαo subunit of GPCR	De novo	Infancy to childhood	Developmental delay and ID Long-lasting MD exacerbations not related to sleep Epilepsy can be absent or well controlled
*GRIN1*	Mental retardation, autosomal dominant 8	GluN1 subunit of NMDAR	De novo/AR	Infancy	Severe developmental delay and ID Early-onset epileptic seizures Oculogyric crises Cortical blindness, dysmorphic traits, microcephaly
*FRRS1L*	Early infantile epileptic encephalopathy-type 37	Ferric Chelate Reductase 1-like	AR	Infancy	Psychomotor regression after normal development Severe encephalopathic epilepsy Choreo-athetosis in infancy/childhood, parkinsonism in adolescence
*TBC1D24*	Early infantile epileptic encephalopathy type 16	TBC1 domain family, member 24	AR	Infancy	Early-onset myoclonic seizures Variable degrees of ID Dystonia
*GPR88*	*GPR88-*related chorea	G protein-coupled receptor 88	AR	Infancy to childhood	Developmental and language delay Severe mental retardation Scarcely progressive chorea
*KMT2B*	DYT28 dystonia	lysine-specific histone methyltransferase 2B	De novo/AD	Childhood-adolescence	Onset in lower limbs and prominent oro-mandibular/laryngeal involvement Mild dysmorphic traits; mild ID Good and sustained response to pallidal DBS
*ATP1A3*	AHC RDP CAPOS syndrome	Na^+^/K^+^ ATPase, α3 subunit	De novo/AD	Infancy to fifth decade	Abrupt onset of neurological signs (dystonia, muscular weakness, ataxia) Initial hemisomatic distribution Identifiable triggering factors

MD, movement disorders; GPCR, guanine nucleotide-binding protein-coupled receptors; NMDAR, glutamatergic *N*-methyl-D-aspartate receptors; ID, intellectual disability; AHC, alternating hemiplegia of childhood; RDP, rapid-onset dystonia parkinsonism; CAPOS, cerebellar ataxia, areflexia, pes cavus, optic atrophy, and sensorineural hearing loss; DBS, deep brain stimulation

## Complex Hyperkinetic Movement Disorders Without Epilepsy as Core Feature

### *ADCY5-*Related Disorders

The first pathogenic dominant mutation in *ADCY5* was identified in a large kindred of German descent initially described in 2001 by Fernandez et al. [2•, 3]. Affected subjects showed an early-onset hyperkinetic movement disorder initially named familial dyskinesia with facial myokimia (FDFM). Subsequently, mutations in this gene were found in patients with childhood-onset chorea and dystonia, as well as in patients with a non-progressive condition resembling benign hereditary chorea (BHC) who tested negative for *NKX2-1* mutations [4, 5•]. So far, 70 genetically confirmed cases belonging to 45 different families have been reported [2–4, 5•, 6••, 7–14, 15•, 16]. Since the first description, the phenotype associated to *ADCY5* mutations has largely broadened and consequently, the original term FDFM has been replaced by a more comprehensive definition (*ADCY5*-related dyskinesias). Patients present virtually in all cases with axial hypotonia and delayed motor and/or language milestones during infancy, associated with early-onset chorea with a generalized distribution, classically involving also the facial muscles and the perioral region [5•, 15•]. Dystonic posturing of the limbs and myoclonic jerks can be prominent, mimicking a myoclonus-dystonia-like phenotype but without the classical upper body distribution observed in *SGCE* mutation carriers [15•, 17]. Episodic exacerbations of movement disorder lasting up to hours have been described in most *ADCY5*-positive cases, being frequently not only related to sleep but also triggered by febrile illnesses and other various stressors [18]. Such episodes can precede the onset of the chronic movement disorder that eventually dominate patients’ clinical picture [15•]. Pyramidal signs in the lower limbs and dysarthria are common clinical findings; in single kindred, dilated cardiomyopathy was reported to co-segregate with *ADCY5* mutations in affected individuals, but this finding has never been observed in other families [3]. Both dominant families and sporadic cases due to de novo mutations have been published, with a recurrent missense mutation (p.Arg418Trp) reported in the majority the affected cases. Two additional mutations at amino acidic residue 418 (p.Arg418Gln and p.Arg418Gly) have been subsequently reported, thus indicating that arginine 418 is a mutational hot spot with a relevant pathogenetic role [6••, 8]. The severity of the movement disorder and the consequent degree of functional disability are variable in affected subjects. The available evidence suggests that the p.Arg418Trp mutation is responsible for a more severe clinical picture, whereas the p.Ala726Thr mutation is associated with a milder phenotype [6••, 15•]. Besides genotype-phenotype correlation, somatic mosaicism detected in some mildly affected patients further explains the clinical heterogeneity of *ADCY5* mutated subjects [5•, 6••].

In terms of therapy, anticholinergics, benzodiazepines (clonazepam), tetrabenazine, baclofen, neuroleptics, and anticonvulsants alone or in combination have been administered to reduce dyskinesias, with variable response; acetazolamide, a carbonic anhydrase inhibitor, has been successfully used in two patients [2•]. Deep brain stimulation (DBS) of bilateral globus pallidus interna (GPi) has been performed in four patients, with moderate improvement of chorea [9, 11].

From a disease mechanism point of view, *ADCY5* encodes adenyl cyclase 5 (AC5), an enzyme most abundantly expressed in striatal neurons that converts adenosine triphosphate (ATP) to cyclic adenosine monophosphate (cAMP). Importantly, dopamine and adenosine modulation of striatal medium spiny neurons (MSNs) is largely mediated through cAMP signaling, as AC5 activity is promoted by the stimulation of the G protein-coupled dopamine receptors type 1 and adenosine receptors 2A [19].

### PDE10A-Related Disorders

Both dominant and recessive mutations in this gene have been recently reported in patients with childhood-onset chorea with a generalized distribution and also involving the facial muscles. Two recurrent dominant mutations (p.Phe300Leu and p.Phe334Leu) have been found so far in five unrelated patients worldwide, arising de novo in all but one subject, whose family showed a dominant pattern of inheritance with complete penetrance [20••, 21, 22]. Recessive homozygous mutations (p.Tyr107Cys and p.Ala116Pro) have been detected in eight patients from two consanguineous pedigrees [23••]. Clinically, carriers of dominant mutations display a homogeneous phenotype characterized by early onset (5–15 years) chorea, normal development and cognition, and characteristic symmetrical T2-hyperintense bilateral striatal lesions on brain MRI. Disease course seems to be non-progressive although diurnal fluctuations in childhood and a progressive spreading of chorea during life with increased severity in the elderly have been reported [20••, 21]. Also, levodopa-responsive parkinsonism with abnormal DAT-scan has been described in an adult positive patient [20••]. However, more evidence is needed to establish whether this is truly part of the *PDE10A*-realted phenotype in the elderly or a chance association. Patients carrying biallelic *PDE10A* mutations show a more severe phenotype, with markedly delayed motor and language milestones, axial hypotonia, an earlier age at onset of chorea (within 6 months of age), severe dysarthria, and mild ID in some cases. In a single affected subject, childhood-onset epilepsy was also reported. Despite a more severe neurological involvement, the MRI of these cases is normal, without striatal abnormalities observed in cases with dominant mutations [23••].

*PDE10A* encodes phosphodiesterase 10A, which regulates the degradation of cAMP and cGMP in MSNs of the corpus striatum, where it is highly and selectively expressed. Both recessive and dominant mutations in *PDE10A* have been shown to lead to a loss of enzymatic function or reduced striatal protein levels [20••, 23••].

Interestingly, preliminary evidence suggests that pathogenic mutations in *ADCY5* may act through a gain of function mechanism [4], overall supporting the hypothesis that abnormally increased levels of intracellular cAMP in striatal neurons may represent a key mechanism in the pathogenesis of chorea.

### KMT2B-Related Disorders

*KMT2B*, also known as *MLL4*, encodes a ubiquitously expressed histone lysine methyltransferase involved in methylation of histone H3 at lysine 4 (H3K4). This gene, located on the chromosomal region 19q13.12, belongs to the SET/MLL family of proteins, which are essential for activating specific sets of genes during normal development [24]. Consistently, loss-of-function mutations in other MLL-encoding genes have been reported in a number of human developmental disorders, such as Kabuki syndrome [25]. Mutations in *KMT2B*, including interstitial microdeletions detected by microarray at 19q13.11-19q13.12, have been reported in patients with childhood-onset dystonia with a progressive course and a variable number of additional clinical features [26••, 27••]. Patients classically present with lower limb dystonia in early childhood, with subsequent generalization as observed in DYT1 mutation carriers; however, unlike DYT1 patients, affected subjects develop a prominent oro-mandibular and laryngeal involvement that can lead to severe dysarthria or even anarthria. Most cases carry de novo dominant mutations, but a limited number of families with an autosomal dominant transmission have been reported as well [27••, 28]. Intrafamilial clinical heterogeneity, with variable severity of dystonia as well as incomplete penetrance, either true o due to possible parental mosaicism have been observed [26••, 27]. So far, 33 unrelated patients carrying *KMT2B* variants with convincing evidence of pathogenicity have been reported, of which 19 carried genomic microdeletion involving *KMT2B* and different contiguous genes [16, 26••, 27••, 28]. *KMT2B*-related dystonia has been defined “complex” in that additional neurological and systemic features have been recognized in some of the mutation carriers, including psychomotor and language delay, minor dysmorphic traits and a characteristic facial appearance (bulbous nasal tips and elongated face), mild-to-moderate ID, short stature, skin abnormalities, and psychiatric disturbances [26••]. In some patients, the complexity of the clinical phenotype could be partially related to the extension of microdeletions on chromosome 19 leading to haploinsufficiency of a variable number of genes contiguous to *KMT2B*. Notably, a marked improvement of dystonia with sustained clinical benefit on long-term follow-up has been reported following bilateral GPi DBS, whereas no oral medication is reported to be particularly effective in alleviating motor manifestations [26••, 27••, 28].

Meyer et al. reported a detection rate of *KMT2B* mutations in up to 38% of patients with early-onset progressive dystonia, suggesting that the contribution of this recently discovered gene to the pathogenesis of childhood-onset dystonia is far higher than most other dystonia-related genes [26••].

### ATP1A3-Related Disorders

*ATP1A3* gene encodes the α3 isoform of the catalytic subunit of the Na^+^/K^+^ pump, which is an adenosine triphosphatase (ATPase) cation transporter playing a crucial role in maintaining electrochemical gradients for Na^+^ and K^+^ across the plasma membrane of different cellular types [29]. Mutations affecting the α3 subunit, which is selectively expressed in neurons, were initially linked to three distinct neurological phenotypes, including rapid-onset dystonia parkinsonism (RDP), alternating hemiplegia of childhood (AHC), and cerebellar ataxia, areflexia, pes cavus, optic atrophy, and sensorineural hearing loss (CAPOS) syndrome [30–33]. Other presentations that do not fall within these neurological entities, as well as intermediate and overlapping phenotypes, have emerged in recent years, providing evidence that these conditions are different manifestations of a wide phenotypic spectrum rather than allelic disorders [34•, 35].

Mutations in *ATP1A3* arise de novo in most cases of AHC, whereas autosomal dominant transmission has been documented in RDP and CAPOS syndrome cases; moreover, germline mosaicism has been recently reported in two families with recurrence of AHC in offspring of unaffected parents [35, 36].

In AHC, symptoms begin before 18 months, and developmental delay is the rule. Clinical manifestations consist of paroxysmal episodes of unilateral hemiplegia or quadriplegia, dystonia, or oculomotor abnormalities (such as monocular nystagmus) which disappear upon sleeping, sometimes only transiently. Episodes last from minutes to several days and can occur with a variable frequency, up to multiple times a day. About half of cases develop epilepsy [37].

In RDP, patients present with abrupt onset of asymmetric dystonia with generally minor features of parkinsonism, with a clear rostro-caudal spreading (face > arm > leg) and prominent bulbar involvement. Symptoms evolve over a few minutes to 30 days, with subsequent stabilization within 1 month; disease course is often biphasic, with a sudden second worsening of symptoms during life. Age at onset ranges from infancy to the fifth decade [38].

In AHC and RDP, clinical manifestations are typically triggered by environmental, physical, or emotional stressors (excitement, strong emotions, physical exertion, febrile illness, excessive environmental stimuli—sounds, light, etc.). The recognition of provocative factors triggering paroxysmal neurological symptoms with an initial hemisomatic distribution represents the most pathognomonic feature to diagnose *ATP1A3*-related disorders and must be carefully investigated in the patients’ clinical history [35].

Atypical phenotypes include paroxysms of unresponsiveness, bulbar signs, ataxia, fever-induced encephalopathy, prolonged flaccid tetraplegia with persistent choreo-athetosis between episodes, catastrophic epilepsy, and progressive childhood-onset cerebellar syndrome with step-wise deterioration [34•, 39–41].

From a genetic point of view, mutations are distributed over almost all *ATP1A3* coding sequence, but RPD phenotypes are mainly associated with mutations in exons 8, 14, and 17, whereas the majority of mutations in patients with AHC are located in exons 17 and 18 [34•]. Available evidence supports a genotype-phenotype correlation with mutations causing classic AHC affecting trans-membrane and functional protein domains; two recurrent missense mutations (p.Asp801Asn and p.Glu815Lys) have been detected in 50% of AHC cases reported. Moreover, only four missense variants have been detected so far in AHC-RDP intermediate phenotypes, and a single recurrent missense mutation (p.Glu818Lys) has been identified in all CAPOS cases [34•, 35].

No specific drugs targeting the altered ionic transport across the Na^+^/K^+^ pump are available, and symptomatic treatment of acute attacks mostly with benzodiazepines and other sleep inducers is the most frequent therapeutic approach in AHC. Flunarizine is widely used as a prophylactic agent; in a series of 30 AHC patients, it was effective to reduce frequency and duration of attacks in 50% of cases, but no controlled trials are available [42•]. Topiramate is also used with the same aim based on anecdotal reports. In RDP, treatment of dystonia and parkinsonism does not benefit from dopaminergic drugs, and GPi DBS has proven ineffective in a very limited number of cases and also in the authors’ experience [43].

### GPR88-Related Disorders

Alkufri et al. recently individuated a recessive homozygous truncating mutation (p.Cys291*) in *GPR88* in three affected children from a consanguineous Palestinian family [44•]. Patients (all females) presented with developmental delay in infancy, markedly delayed speech and learning disability followed, around 9 years of age, by chorea initially affecting the facial muscles and subsequently spreading to involve upper limbs (mainly distally), trunk, and tights. Chorea showed a slow but constant progression over a period of months, without further worsening few years after the onset. Severe mental retardation (IQ 40 in one subject) and a scarcely progressive movement disorder therefore seem to be key phenotypic features of this disorder. So far, no additional cases following the original publication have been reported.

The *GPR88* gene encodes a G protein-coupled receptor (GPCR) abundantly expressed both in D1R- and D2R-expressing MSNs, which are, respectively, part of the direct and indirect pathway [45].

MSNs from *GPR88* knock-out mice show increased glutamatergic excitability and reduced GABAergic inhibition, which results in enhanced firing rates in vivo, producing a murine phenotype characterized by hyperactivity, impaired motor coordination, and motor learning [46].

## Hyperkinetic Movement Disorders in Epileptic-Dyskinetic Encephalopathies

Early onset encephalopathies are a heterogeneous group of diseases characterized by severe dysfunction of cognitive, sensory, and motor development. The etiology of these disorders is variable and includes acquired causes such as prematurity, congenital infections and hypoxic insult at birth, as well as various genetic defects that disrupt brain function, or its normal structure and development. Early-onset, often drug-resistant seizures are a recurrent feature of several encephalopathies. In recent years, the co-occurrence of hyperkinetic movement disorders (chorea, dystonia, ballismus, complex stereotypies) in early-onset epileptic encephalopathies (EOEE) has been increasingly recognized and detailed, to the point that movement disorders are now considered a core feature of several EOEE. These conditions, currently referred to as “epileptic-dyskinetic encephalopathies” (MIM: 308350), are clinically and genetically heterogeneous and encompass various degrees of ID and severe, often intractable epilepsy in association with hyperkinetic movement disorders. Altered functioning of glutamatergic NMDA and AMPA receptors as well as impaired neurotransmission and synaptic plasticity in early neurodevelopmental stages seem to be relevant pathogenetic mechanisms that can give rise to a wide spectrum of variably associated neurological symptoms including movement disorders, ID, and epilepsy.

### FOXG1-Related Disorders

*FOXG1* (Forkhead Box G1) gene, a transcription repressor, plays a crucial role in fetal telencephalon development and is an important component of the transcription regulatory network that controls proliferation, differentiation, neurogenesis, and neurite outgrowth in the cerebral cortex, hippocampus, and basal ganglia [47, 48]. Mutations in *FOXG1* cause a distinct developmental encephalopathy manifesting in infancy or early childhood with severe developmental delay, acquired microcephaly, profound ID, epilepsy, and absent language (so called “congenital Rett syndrome”; OMIM 613454) [49]. Corpus callosum hypoplasia or aplasia, delayed myelination, simplified gyration, and fronto-temporal abnormalities are frequent radiological findings. Beyond these core features, the phenotypic spectrum of *FOXG1* mutations has recently expanded to include early-onset, complex hyperkinetic movement disorders featuring various combinations of chorea, dystonia, dyskinesia, myoclonus, and hand stereotypies that are virtually observed in all positive patients and become evident since the first years of life [50]. In a series of 28 patients, chorea was the most frequent movement disorder (88%), followed by orolingual/facial dyskinesia (80%) and dystonia (76%); movement disorder course was progressive in about half of cases, with remarkable severity and disability [51••]. In a recent review of 83 novel and published cases, dyskinesias and hand stereotypies were both reported in 90% of patients whose clinical data were available. A definite genotype-phenotype correlation has not been established, although truncating *FOXG1* mutations in the N-terminal and the forkhead domains (except conserved site 1) are associated with more severe phenotypes, whereas missense variants in the forkhead conserved site 1 seem to be responsible for milder phenotypes, with independent ambulation, spoken language, normal head growth, and ability to use hands [51••, 52].

Several drugs have shown little or no benefit in alleviating *FOXG1* movement disorders, although levodopa, tetrabenazine, and pimozide were partially beneficial in single cases [50, 51••].

Hyperkinetic movements similar to those observed in *FOXG1* have also been reported in carriers of *CDKL5* mutations, transmitted as an X-linked trait. Mutations in this gene are associated with variant Rett syndrome characterized by onset of refractory seizures within the first weeks of life inconstantly associated with movement disorders [53].

The differential diagnosis of *FOXG1*-related phenotypes includes other epileptic-dyskinetic encephalopathies such as *ARX-*related encephalopathy (characterized by infantile spasms, neonatal-onset progressive dystonia with recurrent status dystonicus, and severe mental retardation) and three conditions caused by de novo mutations in genes essential for neurotransmitter release through synaptic vesicle fusion [54–56]. These include mutations in *STXBP1* (featuring infantile-onset epilepsy with good prognosis, tremor, and frequent paroxysmal non-epileptic movement disorders), *SYT1* (associated with severe developmental delay and an early onset, paroxysmal dyskinetic movement disorder worsening at night), and *UNC13A* (linked to developmental and speech delay, ID, dyskinesias, and intention tremor, with febrile seizures as a minor feature) [57–59].

### GNAO1-Related Disorders

*GNAO1* encodes a subclass (Gαo) of the Gα subunit of heterotrimeric guanine nucleotide-binding proteins which is highly expressed in the brain, where it is involved in the regulation of neuronal excitability and neurotransmission. De novo mutations in *GNAO1* were initially associated with Ohtahara syndrome, a severe type of early epileptic encephalopathy characterized by neonatal tonic spasms, severe motor developmental delay, and ID with a suppression-burst pattern on EEG [60, 61]. *GNAO1*-related encephalopathy has been further characterized following the individuation of additional mutation carriers, and hyperkinetic movements have emerged as an important core feature, being universally present in affected subjects. Patients present in most cases a combination of generalized chorea associated with dystonia, which manifest within the first months or years of life, with a median age at onset around 2 years [62••]. Facial and oro-lingual dyskinesia and complex stereotypies have been reported as well. Movement disorders display a chronic course with characteristic episodic exacerbations triggered by high temperature, infections, emotions, and purposeful movements lasting from minutes to days and even months and often being accompanied by dysautonomic manifestations (sweating, tachycardia, hypertemia, diaphoresis) and thus being potentially life-threatening [63]. Exacerbations have a variable frequency and can present in clusters up to several times a day. While developmental delay and severe ID are features consistently associated with *GNAO1* mutations, epilepsy is variably present and often follows the onset of movement disorders of months or years [64•].

So far, 45 genetically proven patients have been reported, harboring 25 different mutations (23 missense, one splice site, one deletion) [62••, 64•, 65–69]. Glutamine at position 246 (Glu246) and arginine at position 209 (Arg209), both highly conserved amino acids, are *GNAO1* mutational hotspots, and missense mutations involving these residues have been reported in about half (21/45, 46.7%) of published cases. A genotype-phenotype correlation has recently been suggested in functional in vitro studies, with *GNAO1* loss-of-function mutations associated with epileptic encephalopathy and gain-of-function or normally functioning alleles leading to phenotypes dominated by movement disorders [70••].

Tetrabenazine and neuroleptics seem to be the most effective drugs to treat movement disorders in *GNAO1* mutation carriers; in severe drug-resistant cases, bilateral GPi DBS has significantly improved the frequency and severity of exacerbations as well as patients’ motor performances, although the baseline movement disorder seems to remain rather constant even after DBS implant [66, 71,72].

### GRIN1-Related Disorders

*GRIN1* encodes the GluN1 subunit of the glutamatergic *N*-methyl-D aspartate receptors (NMDAR), which are heteromeric protein complexes acting as ion channels upon ligand activation [73]. GluN1 subunits have a key role in the plasticity of synapses, which underlies memory and learning [74]. De novo heterozygous variants of *GRIN1* were first linked to non-syndromic ID with or without epilepsy [75]. So far, 34 positive patients from 30 different families have been reported [76, 77•, 78–80]. The vast majority of mutations are heterozygous variants arising de novo, but recessive biallelic mutations have also been described in seven patients from three different consanguineous kindred [77•, 78]. *GRIN1* positive patients present in almost all cases with severe developmental delay, cognitive dysfunction, and profound ID since early infancy. About 70% of cases develop early-onset, polymorphic seizures with non-specific EEG patterns that are drug-resistant in about one third of cases. Hyperkinetic movement disorders (mainly a combination of chorea and dystonia) have been observed in about 60% of patients, and complex stereotypies as well as oculogyric crises resembling those of monoamine neurotransmitter disorders are frequently reported, being an important diagnostic clue [77•]. Additional features include spastic tetraparesis, cortical blindness, non-specific sleep disturbances, subtle dysmorphism, and microcephaly. All de novo *GRIN1* mutations cluster within or in close proximity of the trans-membrane domains of GluN1, a highly-conserved region; in vitro studies demonstrated that variants in this position lead to a dominant negative effect, whereas one of the reported homozygous variants (c.649C>T; p.Arg217Trp) causes impaired activation of the NMDA receptor [77•]. Moreover, a *GRIN1* truncating variant (c.1666C>T; p.Gln556*), resulting in GRIN1 haploinsufficiency, seems to be tolerated in a heterozygous state, not producing a neurological phenotype; however, when in homozygosity, it has proven responsible for a fatal neonatal epileptic encephalopathy [77•].

### FRRS1L-Related Disorders

The *FRRS1L* gene encodes a component of the outer core of AMPA receptor accessory proteins. Glutamatergic AMPA receptors represent the most common receptor subtype in the brain, mediating fast glutamatergic excitatory post-synaptic potentials. Using a combination of homozygosity mapping and WES, Madeo et al. recently identified four different homozygous mutations in *FRRS1L* in eight patients from four different pedigrees, two of which were consanguineous [81••]. Six additional patients from a large consanguineous Arab kindred have subsequently been reported [82]. Affected subjects present around 20 months of age with psychomotor regression after a phase of normal development, followed by the onset of progressive choreo-atethosis and ballismus and severe encephalopathic epilepsy. Differently from *GNAO1* positive patients, the severity of movement disorders seems to decrease over disease course, giving way to an akinetic-rigid phenotype in late adolescence, and no episodic exacerbations have been reported [81••].

### TBC1D24-Related Disorders

*TBC1D24* is involved in synaptic vesicles trafficking and is expressed in multiple human tissues, with the highest expression in the brain [83]. Recessive homozygous or compound heterozygous mutations in *TBC1D24* have been linked to several human diseases, ranging from non-syndromic deafness to a wide spectrum of epilepsies, whereas dominant mutations have been linked to a type of non-syndromic hearing loss. The most common epilepsy phenotype consists of early-onset myoclonic epilepsy, myoclonic seizures (often occurring in clusters), and drug-resistance. About 50 epileptic patients carrying *TBC1D24* have been reported worldwide, with heterogeneous presentations and prognosis [84•]. In a recent review of new and published cases, 39/48 (81%) presented mild to profound intellectual disability, which therefore appears to be a frequently encountered clinical feature. Moreover, dystonia (sometimes with a hemisomatic distribution) was reported in 7/48 (14.5%) of patients as well as in a recently published case with a complex phenotype including epilepsy, infantile-onset parkinsonism, cerebellar signs, and psychosis [85]. Cortical myoclonus affecting lower limbs with gait impairment has also been reported [86].

## Conclusions

In the last 5 years, the list of genes associated with dystonia, chorea, myoclonus, and mixed movement disorders has dramatically expanded and so has the phenotype associated with mutations in individual genes.

Our systematic review of the recent literature shows that an unexpected variety of molecular causes underlie complex hyperkinetic disorders. Several genes, though individually very are, can be responsible for the same phenotypes and, on the other hand, mutations in a given gene can be associated with several phenotypes, which are often part of spectrum and not discrete entities (as exemplified by *ATP1A3*-related disorders). With some exceptions (e.g., *ADCY5*-, *KMT2B*-, *ATP1A3*-related movement disorders), the number of reported patients affected by these novel genetic entities is still rather limited. Therefore, our knowledge about the clinical features and natural history of these disorders will only grow once larger case series will become available.

Importantly, NGS is challenging the traditional—and often problematic—approach to patients with hyperkinetic movement disorders, based on the recognition of “core clinical features.” The increasing availability of NGS in clinical practice will hopefully help to formulate definite diagnoses in a larger number of patients affected by complex movement disorders, allowing clinicians to provide families with appropriate genetic counseling and disease-specific therapies.
